# Update on the Impact of Depot Medroxyprogesterone Acetate on Vaginal Mucosal Endpoints and Relevance to Sexually Transmitted Infections

**DOI:** 10.1007/s11904-023-00662-0

**Published:** 2023-06-21

**Authors:** Smritee Dabee, Christina Balle, Maricianah Onono, Steve Innes, Gonasagrie Nair, Thesla Palanee-Phillips, Adam D. Burgener, Steven E. Bosinger, Jo-Ann S. Passmore, Renee Heffron, Heather Jaspan, Anna-Ursula Happel

**Affiliations:** 1 Center for Global Infectious Disease, Seattle Children’s Research Institute, 307 Westlake Ave. N, Seattle, WA 98109 USA; 2 Department of Pathology, University of Cape Town, Anzio Road, Observatory, Cape Town, 7925 South Africa; 3 Institute of Infectious Disease and Molecular Medicine, University of Cape Town, Anzio Road, Observatory, Cape Town, 7925 South Africa; 4 Kenya Medical Research Institute, Busia Rd, Kisumu, Kenya; 5 Desmond Tutu Health Foundation, 3 Woodlands Rd, Woodstock, Cape Town, 7915 South Africa; 6 Wits Reproductive Health and HIV Institute, University of the Witwatersrand, Klein St & Esselen St, Hillbrow, Johannesburg 2001 South Africa; 7 Center for Global Health and Diseases, Case Western Reserve University, 10900 Euclid Ave., Cleveland, OH 44106 USA; 8 Department of Obstetrics and Gynecology, University of Manitoba, 66 Chancellors Cir, Winnipeg, MB R3T 2N2 Canada; 9 Unit of Infectious Diseases, Department of Medicine Solna, Center for Molecular Medicine, Karolinska Institute, Karolinska University Hospital, Visionsgatan 18, L8, 171 76 Solna, Sweden; 10 ENPRC Genomics Core Laboratory, Emory National Primate Research Center, 954 Gatewood Rd NE, Atlanta, GA 30329 USA; 11 Department of Pathology and Laboratory Medicine, Emory University School of Medicine, 2015 Uppergate Dr, Atlanta, GA 30307 USA; 12 Emory Vaccine Center, Emory University, 7 1st Ave, Atlanta, GA 30317 USA; 13 National Health Laboratory Service, Anzio Road, Observatory, Cape Town, 7925 South Africa; 14 Department of Medicine, University of Alabama at Birmingham, 845 19th Street South, AL 35294-2170 Birmingham, USA; 15 Department of Pediatrics, University of Washington, 1959 NE Pacific St., Seattle, WA 98195 USA; 16 Department of Global Health, University of Washington, 1510 San Juan Road NE, Seattle, WA 98195 USA

**Keywords:** Depot medroxyprogesterone acetate, Vaginal microbiome, Inflammation, Immune cells, Epithelial barrier, ECHO Trial

## Abstract

**Purpose of Review:**

The long-acting reversible intramuscularly-injected contraceptive depot medroxyprogesterone acetate (DMPA-IM) is widely used by cisgender women in Africa. Although DMPA-IM provides reliable contraception, potential effects on the female genital tract (FGT) mucosa have raised concern, including risk of HIV infection. This review summarises and compares evidence from observational cohort studies and the randomised Evidence for Contraceptive Options in HIV Outcomes (ECHO) Trial.

**Recent Findings:**

Although previous observational studies found women using DMPA-IM had higher abundance of bacterial vaginosis (BV)-associated bacteria, increased inflammation, increased cervicovaginal HIV target cell density, and epithelial barrier damage, sub-studies of the ECHO Trial found no adverse changes in vaginal microbiome, inflammation, proteome, transcriptome, and risk of viral and bacterial STIs, other than an increase in Th17-like cells.

**Summary:**

Randomised data suggest that DMPA-IM use does not adversely change mucosal endpoints associated with acquisition of infections. These findings support the safe use of DMPA-IM in women at high risk of acquiring STIs, including HIV.

**Graphical Abstract:**

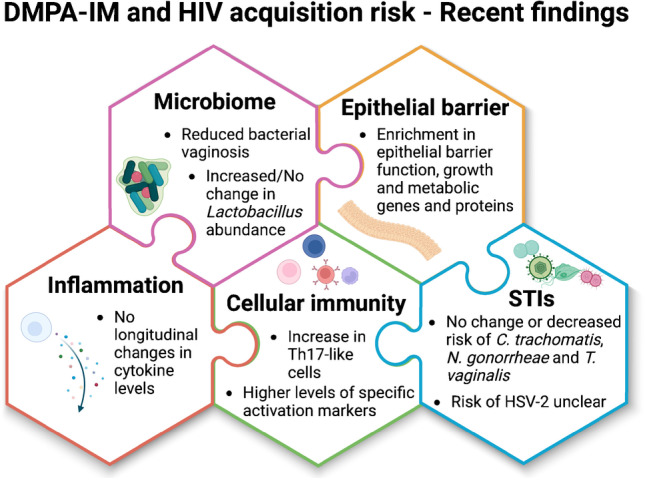

## Introduction

Identifying safe hormonal contraceptive options is an important public health priority, particularly for cis-women in Sub-Saharan Africa, as they are at high risk of both vaginal infections and unintended pregnancies. Uptake of the long-acting reversible intramuscularly injected contraceptive depot medroxyprogesterone acetate (DMPA-IM) has increased over the past decades [1] and is currently the most common contraceptive used in Sub-Saharan Africa [2].

Systematic reviews and meta-analyses synthesising results from >30 observational studies concluded that there was epidemiological, clinical, and laboratory evidence that DMPA-IM use was associated with 40–50% increased risk of HIV acquisition, although findings from observational studies had been inconsistent and likely biased by sexual behaviour [3–6]. In order to address some of the biases and to attempt to assess causality, the open-label Evidence for Contraceptive Options and HIV Outcomes (ECHO) Trial randomised approximately 7800 contraceptive-seeking women in a multicentre, open-label trial across 12 research sites in eSwatini, Kenya, South Africa, and Zambia in a 1:1:1 ratio to DMPA-IM, a copper intrauterine device (IUD) or levonorgestrel (LNG) subdermal implant, all of which are highly effective, reversible, long-acting contraceptives [7]. The study did not find a substantial difference in HIV risk amongst the methods evaluated, being powered to detect a 50% increase in HIV incidence across study arms [7]. The HIV incidence was 4.19 per 100 woman-years [3.54–4.94] in the DMPA-IM group, 3.94 per 100 woman-years [3.31–4.66] in the Cu-IUD group, and 3.31 per 100 woman-years [2.74–3.98] in the implant group. In a modified intention-to-treat analysis, the hazard ratios (HRs) for HIV acquisition were 1.04 (96% CI 0.82–1·33, *p*=0.72) for DMPA-IM compared with Cu-IUD users, and 1.23 (0.95–1.59, *p*=0.097) for DMPA-IM compared with LNG implant users [7]. An updated systematic review concluded that the additional HIV incidence data from the randomised ECHO Trial does not support previous concerns that DMPA-IM use increases the risk of HIV acquisition [8••]. Given the outcome of the ECHO trial, the WHO updated their guidance on contraceptive use and recommended no restriction on use of any reversible hormonal contraceptive method for women at high risk of HIV acquisition.

Like previous reports on associations between DMPA-IM use and HIV risk, observational studies have provided conflicting evidence about the relationship between DMPA-IM use and genital mucosal factors associated with HIV acquisition. Thus, sub-studies of the ECHO Trial have evaluated these mucosal endpoints, including the vaginal microbiome, cervicovaginal inflammation, immune cell populations, proteome, epithelial barrier function, and sexually transmitted infection (STI) acquisition risk [9••, 14,10••, 11••, 12••, 13••, ]. Here, we summarise recent observational and randomised data to provide an update on the effect of DMPA-IM on vaginal mucosal endpoints relevant to risk of acquiring genital infections.

## Mucosal Endpoints Associated with HIV Acquisition

### Vaginal Microbiome

Bacterial vaginosis (BV) is a common dysbiosis of the vaginal microbiome of women of reproductive age, identified using Amsel criteria (that evaluates a clinical array of diagnostic criteria) or Nugent Scoring (which captures bacterial morphotypes on Gram stain) [15]. BV defined by these algorithms often overlaps with a lower female genital tract (FGT) microbiota defined by higher bacterial diversity using molecular sequencing, depleted levels of beneficial *Lactobacillus* spp., and higher abundances of anaerobic species, including *Gardnerella vaginalis*, *Fannyhessea vaginae* (previously *Atopobium vaginae*), and *Prevotella bivia* [15]. Large cohort studies and meta-analyses have consistently found associations between BV (diagnosed by Amsel criteria or Nugent scoring) and increased risk of HIV acquisition [16, 17]. Using 16S rRNA gene sequencing, women with specific bacterial community state types (CSTs), particularly CST-IV (highly diverse, dominated by a range of facultative and/or obligate anaerobes and lack of *Lactobacillus* spp.), are at an increased risk of HIV acquisition [18]. A higher relative abundance of specific bacterial taxa has also been associated with HIV acquisition, including *Prevotella melaninogenica*, *Prevotella bivia*, *Prevotella ihumii*, *Veillonella montpellierensis*, *Mycoplasma* spp., *Sneathia sanguinegens*, and TM7-H1 [13••, 18]. In contrast to relative abundance data, which can vary significantly amongst women based on their microbiota composition and presence of specific taxa, quantitative PCR (qPCR) allows the assessment of absolute concentrations of bacterial species. McClelland et al. found that absolute concentrations of *Parvimonas* species types 1 and 2, *Gemella asaccharolytica*, *Mycoplasma hominis*, *Leptotrichia/Sneathia*, *Eggerthella* species type 1, and *Megasphaera* were associated with HIV risk, in a concentration-dependant manner [19]. Thus, while clinical and molecular methods generally overlap in their description of associations between BV and HIV risk, molecular studies have disagreed with one another on the exact composition of BV-associated bacteria that predict the strongest risk, which is likely partly explained by the differences in the molecular and computational methods applied in molecular analyses.

Several observational studies have suggested that DMPA-IM users are at lower risk of developing BV (by Nugent scoring or Amsel criteria) compared to women not using any hormonal contraceptives [20–25]. In agreement, increases in *Lactobacillus* species or reductions in BV-associated bacteria have been reported in some longitudinal studies that have measured microbiota fluctuations (assessed by qPCR and bacterial culture) before and after DMPA-IM initiation [26–29]. In contrast, others found no change in the absolute concentrations of the BV-associated species *G. vaginalis* and *F. vaginae* measured by qPCR after DMPA-IM initiation, despite a significant decrease in Nugent score [30]. These findings suggest a beneficial shift in the vaginal microbiota composition with DMPA-IM use. Similarly, the vaginal microbiotas of DMPA-IM users in the Vaginal Human Microbiome Project were less likely to have vaginal microbiotas dominated by BV-associated bacteria (measured by 16S rRNA gene sequencing) compared to women using barrier contraception, although this was not associated with a concurrent increase in the relative abundance of *Lactobacillus* species [31].

In contrast, other longitudinal studies reported decreases in vaginal *Lactobacillus* species after women initiate DMPA-IM, as measured by absolute concentrations [32], relative abundances [33], or proportion of women with lactobacilli present by culture [34]. DMPA-IM use correlated with an increased diversity of the vaginal microbiota, assessed by 16S rRNA gene sequencing, in a cross-sectional cohort of Kenyan women [35]. This discrepancy in observational studies does not allow for robust conclusions on the effect of DMPA-IM use on the cervicovaginal microbiota, as many of the reported outcomes could be a result of behavioural differences between women choosing DMPA-IM over other or no contraceptive methods. Randomising women to contraceptive methods is the best method we have to attenuate the confounding effect of behaviour (or any other socio-demographic differences between groups), but this is not perfect, as participants are not blinded to their contraceptive arm.

A sub-study of the ECHO Trial conducted by this team specifically assessed the effect of DMPA-IM initiation on the lower FGT microbiota, compared to the LNG-implant and copper IUD. Brown et al. found that overall bacterial diversity did not significantly increase up to 6 months following DMPA-IM initiation compared to baseline [13••]. Instead, women randomised to DMPA-IM regularly transitioned from *L. iners-* (CST-III) to *L. crispatus-* (CST I-B) dominated communities during the first 6 months of use. Total bacterial 16S gene copies increased significantly in women randomised to DMPA-IM, and this increase was predominantly due to an increase in *Lactobacillus* spp. bacterial load [13••]. In another study that randomised 97 Malawian women with or without HIV to DMPA-IM or LNG-implant and followed them over 6 months, no long-term changes in the genital microbiota were observed with DMPA-IM use in women with HIV and without HIV [36]. Overall, these randomised studies suggest that DMPA-IM use is not associated with non-optimal cervicovaginal microbiota composition that previously has been associated with increased risk of HIV acquisition.

### Cytokines

Inflammatory responses in the FGT are important to fight invading pathogens. However, increases in specific cervicovaginal cytokine concentrations, independent of the cause, have also been associated with increased risk of later HIV acquisition [37–41]. While there is some heterogeneity between studies and populations, higher vaginal fluid interleukin (IL)-1α, IL-1β, and IL-8 concentrations appear to be associated with increased risk of subsequent HIV acquisition in the majority of studies [42].

Several observational cohorts studies that have included Sub-Saharan African women found DMPA-IM use to be associated with changes in cervicovaginal cytokines, with higher IL-1α, IL-1β, macrophage inflammatory proteins (MIP)-1α, MIP-1β, IL-6, IL-8, interferon (IFN) gamma-induced protein 10 (IP-10), RANTES, C-X-C motif chemokine ligand (CXCL)-6, and CXCL-1 [43–46], while other studies, including longitudinal studies analysing the pre- and post-DMPA-IM initiation cytokine levels, have found DMPA-IM to be immunosuppressive, with decreases in IL-1β, IFN-α, IFN-g, IL-6, IL-8, IL-10, IL-12p40, IL-1 receptor antagonist (RA), CXCL-10, monocyte chemotactic protein-1 (MCP-1), granulocyte-colony stimulating factor (G-CSF), and IP-10 [20, 26, 47–53], possibly in a dose-dependent manner [54], and others found no significant differences in cytokine concentrations between DMPA-IM users and those not using any hormonal contraception in a cross-sectional study [55]. Thus, findings of observational studies describing changes in cervicovaginal cytokines and DMPA-IM use were variable.

Cervicovaginal cytokine levels were evaluated in two independent sub-studies that included samples of women from different sites (Cape Town and Johannesburg, South African and Kisumu, Kenya [9••] and Tshwane and eThekwini, South Africa [56••]) of the randomised ECHO Trial [9••, 56••]. Both studies found no significant differences in cytokine levels 1, 3, or 6 months after DMPA-IM initiation compared to pre-DMPA-IM initiation levels. These randomised data support the conclusion that DMPA-IM use is not associated with changes in inflammatory markers relevant to HIV acquisition risk in the lower FGT.

### Immune Cell Populations

The number, phenotype, and activation status of genital tract immune cells are an important determinant of risk of acquisition [57]. Cervicovaginal-activated CD4 + T cells that express the HIV co-receptor CCR5, particularly Th17 (CCR6 +) cells, are preferential targets for HIV infection and have consequently been used as a proxy measure for HIV acquisition risk [10••, 58, 59].

Association between DMPA-IM use and increased numbers of cervical immune cells, including CD4+ T-cells, and increased expression of the HIV co-receptor CCR5 have been described in some observational studies [20, 53, 60–63], but not others [34, 51, 52]. In the longitudinal ZimCHIC study, DMPA-IM initiation led to minimal reductions in the number of cervical CD4+ T cells and CD11c+ antigen presenting cells [52]. Two recent longitudinal studies found significant increases in the frequencies of cervical T cells expressing CCR5 after DMPA-IM initiation using both flow cytometry and imaging [50, 64]. In a South African cohort, women using long-term injectable progestins including DMPA-IM had higher frequency and density of CCR5+CD4+ cervical T cells, versus women not using contraception, but these differences may at least partially be due to differences in sexual behaviour [55]. Other phenotypic alterations of cervical T cells have been observed amongst DMPA-IM users including increased expression of the activation marker HLA-DR and a decreased frequency of regulatory [51] and central memory T cells [50]. Since all these cells are potential HIV target cells, the observational data regarding the effect of DMPA-IM on HIV target cell availability in the cervix have been unclear.

From this group, which included a subset of South African women enrolled in the ECHO Trial, randomisation to DMPA-IM was associated with higher frequencies of cervical Th17-like cells at 1 month after initiation, including a highly susceptible, activated population co-expressing CD38, CCR5, and α4β7, while overall frequencies of CD4+ T cells remained stable [10••]. After 1 month, women using DMPA-IM had significantly more Th17-like cells than women using the Cu-IUD or LNG-implant. This finding is supported by RNA-Seq data that showed that DMPA-IM enriched for pathways associated with T cell responses, specifically of genes that function in T cell activation, migration, and communication (Gupta et al., submitted). Interestingly, the increased abundance of Th17-like cells amongst DMPA-IM users in the ECHO Trial coincided with increased expression of mucosal barrier proteins [10••]. Overall, these randomised data suggest that DMPA-IM initiation may cause increases in cervicovaginal activated or CCR5-expressing CD4 + T cells important for HIV acquisition risk, yet the clinical relevance of these findings is unclear as the changes in HIV target cell frequencies and activation could be counteracted by improved barrier function and not result in increased HIV risk.

### Epithelial Barrier Function

The integrity of the physical barrier of the genital tract is crucial for the defence against HIV and other pathogens. Thinning of the vaginal epithelium or decreased cell adhesion and tight junction proteins leads to a more permeable epithelium and can be used as a measure for epithelial barrier function [65]. Epithelial barrier modulation by DMPA-IM use has been reported using various techniques, including by assessing structure and protein location of biopsies by microscopy or molecular techniques measuring the expression of functional genes or proteins in cross-sectional and prospective studies.

Several cross-sectional studies have identified proteomic indications of vaginal epithelial barrier disruption amongst DMPA-IM users in FGT secretions, including reduced levels of the growth factors G-CSF and M-CSF, which may suggest epithelial damage [45, 49, 66–68]; increased levels of haemoglobin; decreased levels of epithelial repair, maintenance, and structural proteins [69]; and reduced levels of protease inhibition proteins, including matrix metalloproteinases (MMPs) and tissue inhibitors of metalloproteinases (TIMPs) [48, 49, 69, 70].

In a non-randomised prospective study before and after 6 weeks of DMPA-IM initiation versus combined oral contraceptive use, whole-genome transcriptome profiling of the ectocervical mucosa demonstrated that DMPA-IM use resulted in downregulation of genes encoding cell junction proteins involved in epithelial integrity and differentiation, but demographics differed significantly between DMPA-IM and oral contraceptive users which may have confounded these results [71]. Similarly, assessment of ectocervical biopsy tissue from women before and 1 month after initiating DMPA-IM revealed that DMPA-IM use resulted in a decreased expression of the cell-cell adhesion molecule desmosomal cadherin desmoglein-1 (DSG1) and significantly increased mucosal tissue permeability [72]. In a cross-sectional study, expression of genes involved with necrosis was increased in cervical transformation zone tissue, while expression of genes involved with cell proliferation was decreased in women using DMPA-IM versus controls not using any hormonal contraceptive [73]. In contrast to these findings, the total thickness of the ectocervical epithelial layer was comparable between women using DMPA-IM and those not using any hormonal contraceptive in a recent cross-sectional study in Kenyan women [74]. The same observations were made for the vaginal epithelium of Brazilian women [75], while in Swedish women, the vaginal epithelial thickness of DMPA-IM users was greater compared to non-hormonal contraceptive users [61]. In longitudinal studies in women from the USA and Dominican Republic, the number of cell layers and density of E-cadherin, a cell adhesion protein, was comparable prior to DMPA-IM initiation and 6 weeks thereafter [20]. Similarly, no significant change in epithelial thickness, number of cell layers, and E-cadherin and ZO-1, another tight junction protein, was observed after 12 weeks of DMPA-IM use in women from the USA [63], nor after 12 months with regard to epithelial cell layer number [34]. These results from observational studies suggest that epithelial thickness is not influenced by DMPA-IM use, while cell-cell adhesion molecules might be adversely affected.

In a sub-study of the ECHO Trial, RNA-Seq of cervical cytobrushes from women randomised to DMPA-IM revealed a significant enrichment of genes regulating tight junctions in epithelial barriers, including several claudins and junctional adhesion molecules at 1 month post DMPA-initiation versus baseline, but not amongst LNG-Implant and Cu-IUD users (Gupta et al., submitted). Consistent with this, DMPA-IM induced Th17 cells which positively correlated with proteins involved in mucosal barrier integrity, including epithelial cell-cell adhesion junction proteins, protease inhibition, actin cytoskeletal components, and cell-matrix adhesion [10••]. These results contrast previous observational studies reporting downregulation of genes involved in epithelial barrier function. The biological implications of these findings with regard to genital mucosal health are yet unclear. As reviewed elsewhere, tight junction proteins are expressed at higher levels following intestinal epithelial barrier damage, to induce tight junction closure during repair of acute injury [76] but whether the same would occur after vaginal epithelium damage needs to be investigated. Thus, whether the increased expression of tight junction proteins is a result of tissue damage repair or improved barrier function in the FGT remains unknown.

Gupta et al. also found that concurrently with the enrichment of genes regulating tight junctions, gene pathways associated with growth and metabolism (e.g. glycolysis, fatty acid, and protein metabolism) were downregulated amongst DMPA-IM users. Glycosylation of cervicovaginal fluid of the vaginal epithelium provides a barrier to infection, and decreased glycosylation of cervicovaginal fluid proteins could lead to decreased barrier protection [77, 78].

In summary, analyses based on protein and gene expression suggest that DMPA-IM use modulates mucosal barrier function, but it is unclear whether DMPA-IM enhances or impairs barrier function. Differences amongst studies could partially be due to differences in methodological approaches, or could reflect the timing of sampling and thus, the medroxyprogesterone acetate (MPA) concentration. This is supported by findings from the CAPRISA004 study, which showed a dose-dependent effect of DMPA-IM on genital host protein expression [66]. Women with high serum MPA levels had reduced levels of cervicovaginal proteins associated epithelial barrier repair and keratinisation in support of reduced mucosal barrier integrity with DMPA-IM use, while this was not seen for women with lower serum MPA levels [66].

## Bacterial, Protozoal, and Viral STIs Including HIV

Infection with bacterial and protozoal STIs (including *Mycoplasma genitalium*, *Neisseria gonorrhoeae*, *Treponema pallidum* (syphilis), *Trichomonas vaginalis*, and *Chlamydia trachomatis*, and viral STIs, including Herpes simplex virus (HSV-2) and human papillomavirus (HPV)) can increase risk of subsequent HIV acquisition [79–81]. While data evaluating the association between DMPA-IM use and incidence of *M. genitalium* are sparse, a recent systematic review and meta-analysis described a reduced incidence of *T. vaginalis* with DMPA-IM use relative to non-hormonal contraceptive users, and inconclusive evidence for *C. trachomatis*, *N. gonorrhoeae*, and *T. pallidum* [82]*.* A secondary analysis of the randomised ECHO Trial found that DMPA-IM use was associated with a 20% lower risk of *C. trachomatis* infection compared with LNG-implant use and a 30% lower risk of *N. gonorrhoea* infection compared to Cu-IUD use [12••]. *N. gonorrhoea* prevalence was also lower amongst women randomised to DMPA-IM compared to those randomised to LNG-implant, although this was not significant [12••]. These findings, from both observational and interventional studies, suggest that DMPA-IM use is not associated with increased risk of the common bacterial and protozoal STIs *C. trachomatis*, *T. vaginalis*, and *N. gonorrhoea* but more data are needed to make conclusions regarding risk of acquiring *M. genitalium* and *T. pallidum*.

With regard to viral STIs, systematic reviews concluded that the available observational evidence does not support an association between DMPA-IM use and HPV risk [82, 83]. In contrast, two systematic reviews found evidence that DMPA-IM use increases risk of HSV-2 acquisition [82, 83], while there was evidence of a weak protective effect of DMPA-IM use versus Cu-IUD on HSV-2 acquisition in the randomised ECHO Trial [11••]. Given the associations between HSV-2 and subsequent HIV acquisition [11••], the relationship between HSV-2 risk and DMPA-IM use requires further investigation.

## Conclusions

Synthesis of data from the more robust and unbiased studies suggests that DMPA-IM use does not result in notable adverse changes of most of the FGT mucosal endpoints that have been associated with HIV acquisition, including the microbiome, inflammatory markers, and common bacterial and viral STIs, or if anything, is associated with protective changes, except for CCR5-expressing Th17-like cells. Increases in Th17 cells were however accompanied by increases in proteins involved in mucosal barrier function. DMPA-IM users experienced increases in gene pathways involved in junctional proteins, highlighting that the mucosal environment needs to be considered holistically when assessing HIV risk. It needs to be acknowledged that, while reported condom use amongst DMPA-IM users was higher compared to the Cu-IUD and LNG-implant users in the ECHO Trial, data from the biomedical ECHO Trial sub-studies assessing mucosal endpoints associated with HIV risk support the finding that DMPA-IM use is not associated with adverse FGT health. Overall, these new data support the ‘safe’ use of DMPA-IM in all women, also those at high risk for STIs.

## References

[1] Alkema L, Kantorova V, Menozzi C, Biddlecom A. National, regional, and global rates and trends in contraceptive prevalence and unmet need for family planning between 1990 and 2015: a systematic and comprehensive analysis. Lancet [Internet]. 2013;381:1642–52. Available from: https://www.sciencedirect.com/science/article/pii/S0140673612622041. Accessed 28 Nov 2022 10.1016/S0140-6736(12)62204-1 23489750

[2] Tsui AO, Brown W, Li Q. Contraceptive practice in sub-Saharan Africa. Popul Dev Rev [Internet]. 2017;43:166–91. Available from: 10.1111/padr.12051. 10.1111/padr.12051 PMC5658050 29081552

[3] Smith JA, Heffron R, Butler AR, Celum C, Baeten JM, Hallett TB (2017). Could misreporting of condom use explain the observed association between injectable hormonal contraceptives and HIV acquisition risk?. Contraception.

[4] Baeten JM, Heffron R (2015). Contraception and sexually transmitted infections: risks and benefits, hypotheses and evidence. Lancet Glob Health.

[5] Polis CB, Curtis KM, Hannaford PC, Phillips SJ, Chipato T, Kiarie JN, Westreich DJ, Steyn PS. An updated systematic review of epidemiological evidence on hormonal contraceptive methods and HIV acquisition in women. AIDS. 2016;30(17):2665–83. 10.1097/QAD.0000000000001228. 10.1097/QAD.0000000000001228 PMC5106090 27500670

[6] Morrison CS, Chen P-L, Kwok C, Baeten JM, Brown J, Crook AM, et al. Hormonal contraception and the risk of HIV acquisition: an individual participant data meta-analysis. PLoS Med [Internet].; 2015;12:e1001778-. Available from: 10.1371/journal.pmed.1001778. 10.1371/journal.pmed.1001778 PMC4303292 25612136

[7] Ahmed K, Baeten JM, Beksinska M, Bekker L-G, Bukusi EA, Donnell D, et al. HIV incidence among women using intramuscular depot medroxyprogesterone acetate, a copper intrauterine device, or a levonorgestrel implant for contraception: a randomised, multicentre, open-label trial. The Lancet [Internet]. 2019;394:303–13. Available from: 10.1016/S0140-6736(19)31288-7. 10.1016/S0140-6736(19)31288-7 PMC6675739 31204114

[8] •• Curtis KM, Hannaford PC, Rodriguez MI, Chipato T, Steyn PS, Kiarie JN. Hormonal contraception and HIV acquisition among women: an updated systematic review. BMJ Sex Reprod Health [Internet]. 2020;46:8. Available from: http://jfprhc.bmj.com/content/46/1/8.abstract. **This is an updated systematic review on hormonal contraception and HIV acquisition. **Accessed 15 Nov 2022 10.1136/bmjsrh-2019-200509 PMC6978562 31919239

[9] •• Tanko RF, Bunjun R, Dabee S, Jaumdally SZ, Onono M, Nair G, et al. The effect of contraception on genital cytokines in women randomized to copper intrauterine device, depot medroxyprogesterone acetate, or levonorgestrel implant . J Infect Dis [Internet]. 2022;226:907–19. Available from: 10.1093/infdis/jiac084. **This biomedical ECHO sub-study showed that DMPA-IM use does not increase cervico-vaginal cytokines levels at 1- and 6-month post-contraceptive initiation compared to pre-initiation.** 10.1093/infdis/jiac084 PMC9470113 35263421

[10] •• Bunjun R, Ramla TF, Jaumdally SZ, Noël-Romas L, Ayele H, Brown BP, et al. Initiating Intramuscular Depot Medroxyprogesterone Acetate (DMPA-IM) Increases frequencies of Th17-like human immunodeficiency virus (HIV) target cells in the genital tract of women in South Africa: a randomized trial. Clin Infect Dis [Internet]. 2022;ciac284. Available from: 10.1093/cid/ciac284. **This biomedical ECHO sub-study showed that DMPA-IM use was associated with higher frequencies of cervical Th17-like cells at 1-month post-initiation, which as accompanied by enhanced mucosal barrier function as measured by proteomics.** 10.1093/cid/ciac284 PMC9710690 35941737

[11] •• Mugo NR, Stalter RM, Heffron R, Rees H, Scoville CW, Morrison C, et al. Incidence of herpes simplex virus type 2 infection among African women using depot medroxyprogesterone acetate, a copper intrauterine device, or a levonorgestrel implant for contraception: a nested randomized trial . Clin Infect Dis [Internet]. 2022;75:586–95. Available from: 10.1093/cid/ciab1027. **This ECHO sub-study showed that DMPA-IM use was not associated with increased risk of HSV-2 acquisition.** 10.1093/cid/ciab1027 PMC9464069 34910143

[12] •• Deese J, Philip N, Lind M, Ahmed K, Batting J, Beksinska M, et al. Sexually transmitted infections among women randomised to depot medroxyprogesterone acetate, a copper intrauterine device or a levonorgestrel implant. Sex Transm Infect [Internet]. 2021;97:249–55. Available from: https://sti.bmj.com/content/97/4/249. **This ECHO sub-study showed that DMPA-IM use was associated with lower chlamydia and gonorrhoea risk compared to implant and copper-IUD use, respectively.** 10.1136/sextrans-2020-054590 PMC8165154 33208512

[13] •• Brown BP, Feng C, Tanko RF, Jaumdally SZ, Bunjun R, Dabee S, et al. Copper intrauterine device increases vaginal concentrations of inflammatory anaerobes and depletes lactobacilli compared to hormonal options in a randomized trial. Nat Commun [Internet]. 2023;14:499. Available from: 10.1038/s41467-023-36002-4. **This biomedical ECHO sub-study showed that DMPA-IM use was not associated with shifts in bacterial diversity at 1- and 6-months post-contraceptive initiation, and that a shift from*****L. iners*****to*****L. crispatus*****dominated microbiotas at 6-months post-contraceptive initiation was common amongst DMPA-IM users.** 10.1038/s41467-023-36002-4 PMC9886933 36717556

[14] Ayele H, Romas LN, Birse K, Horne S, Onono M, Nair G, Palanee-Phillips T, Tanko R, Bunjun R, Arnold KB, McCorrister S. The impact of progestin-based contraceptive initiation on the cervicovaginal proteome in participants from the ECHO trial. In: Journal of the international aids society. The atrium, southern gate, chichester. England: John Wiley & Sons Ltd; 2021. p. 54.

[15] McKinnon LR, Achilles SL, Bradshaw CS, Burgener A, Crucitti T, Fredricks DN, et al. The evolving facets of bacterial vaginosis: implications for HIV transmission. AIDS Res Hum Retroviruses [Internet]. 2019;35:219–28. Available from: https://www.ncbi.nlm.nih.gov/pubmed/30638028. Accessed 06 Dec 2019 10.1089/aid.2018.0304 PMC6434601 30638028

[16] Atashili J, Poole C, Ndumbe PM, Adimora A, a, Jennifer S. (2008). Bacterial vaginosis and HIV acquisition: a meta-analysis of published studies. AIDS..

[17] Cohen CR, Lingappa JR, Baeten JM, Ngayo MO, Spiegel CA, Hong T, et al. Bacterial vaginosis associated with increased risk of female-to-male HIV-1 transmission: a prospective cohort analysis among African couples. PLoS Med [Internet]. 2012;9:e1001251. Available from: 10.1371/journal.pmed.1001251. 10.1371/journal.pmed.1001251 PMC3383741 22745608

[18] Gosmann C, Anahtar MN, Handley SA, Farcasanu M, Abu-Ali G, Bowman BA, et al. Lactobacillus-deficient cervicovaginal bacterial communities are associated with increased HIV acquisition in young South African women. Immunity [Internet]. 2017;46:29–37. Available from: https://www.ncbi.nlm.nih.gov/pubmed/28087240. Accessed 06 Dec 2019 10.1016/j.immuni.2016.12.013 PMC5270628 28087240

[19] McClelland RS, Lingappa JR, Srinivasan S, Kinuthia J, John-Stewart GC, Jaoko W, et al. Evaluation of the association between the concentrations of key vaginal bacteria and the increased risk of HIV acquisition in African women from five cohorts: a nested case-control study. Lancet Infect Dis [Internet]. 2018;18:554–64. Available from: 10.1016/S1473-3099(18)30058-6. 10.1016/S1473-3099(18)30058-6 PMC6445552 29396006

[20] Thurman A, Chandra N, Schwartz JL, Brache V, Chen BA, Asin S, et al. The effect of hormonal contraception on cervicovaginal mucosal end points associated with HIV acquisition. AIDS Res Hum Retroviruses [Internet]. 2019;35:853–64. Available from: 10.1089/aid.2018.0298. 10.1089/AID.2018.0298 30997816

[21] Jespers V, Crucitti T, Menten J, Verhelst R, Mwaura M, Mandaliya K, et al. Prevalence and correlates of bacterial vaginosis in different sub-populations of women in Sub-Saharan Africa: a cross-sectional study. PLoS One [Internet]. 2014;9:e109670-. Available from: 10.1371/journal.pone.0109670. 10.1371/journal.pone.0109670 PMC4188821 25289640

[22] Haddad LB, Wall KM, Tote K, Kilembe W, Vwailika B, Sharkey T, et al. Hormonal contraception and vaginal infections among couples who are human immunodeficiency virus serodiscordant in Lusaka, Zambia. Obstet Gynecol [Internet]. 2019;134. Available from: https://journals.lww.com/greenjournal/Fulltext/2019/09000/Hormonal_Contraception_and_Vaginal_Infections.20.aspx. Accessed 18 Nov 2022 10.1097/AOG.0000000000003404 PMC9172972 31403592

[23] Bradshaw CS, Vodstrcil LA, Hocking JS, Law M, Pirotta M, Garland SM, et al. Recurrence of bacterial vaginosis is significantly associated with posttreatment sexual activities and hormonal contraceptive use. Clin Infect Dis [Internet]. 2013;56:777–86. Available from: 10.1093/cid/cis1030. 10.1093/cid/cis1030 23243173

[24] van de Wijgert JHHM, Verwijs MC, Turner AN, Morrison CS. Hormonal contraception decreases bacterial vaginosis but oral contraception may increase candidiasis: implications for HIV transmission. AIDS [Internet]. 2013;27. Available from: https://journals.lww.com/aidsonline/Fulltext/2013/08240/Hormonal_contraception_decreases_bacterial.14.aspx. Accessed 18 Nov 2022 10.1097/QAD.0b013e32836290b6 23660575

[25] Vodstrcil LA, Hocking JS, Law M, Walker S, Tabrizi SN, Fairley CK, et al. Hormonal contraception is associated with a reduced risk of bacterial vaginosis: a systematic review and meta-analysis. PLoS One [Internet]. 2013;8:e73055-. Available from: 10.1371/journal.pone.0073055. 10.1371/journal.pone.0073055 PMC3762860 24023807

[26] Roxby AC, Fredricks DN, Odem-Davis K, Ásbjörnsdóttir K, Masese L, Fiedler TL (2016). Changes in vaginal microbiota and immune mediators in HIV-1-seronegative Kenyan women initiating depot medroxyprogesterone acetate. J Acquir Immune Defic Syndr..

[27] Whitney BM, Guthrie BL, Srinivasan S, Tapia K, Muriuki EM, Chohan BH, et al. Changes in key vaginal bacteria among postpartum African women initiating intramuscular depot-medroxyprogesterone acetate. PLoS One [Internet]. 2020;15:e0229586-. Available from: 10.1371/journal.pone.0229586. 10.1371/journal.pone.0229586 PMC7058341 32134931

[28] Borgdorff H, Verwijs MC, Wit FWNM, Tsivtsivadze E, Ndayisaba GF, Verhelst R, et al. The impact of hormonal contraception and pregnancy on sexually transmitted infections and on cervicovaginal microbiota in African sex workers. Sex Transm Dis [Internet]. 2015;42:143–52. Available from: https://www.jstor.org/stable/48511882. Accessed 18 Nov 2022 10.1097/OLQ.0000000000000245 25668647

[29] Kazi YF, Saleem S, Kazi N. Investigation of vaginal microbiota in sexually active women using hormonal contraceptives in Pakistan. BMC Urol [Internet]. 2012;12:22. Available from: 10.1186/1471-2490-12-22. 10.1186/1471-2490-12-22 PMC3492163 22901000

[30] Achilles SL, Austin MN, Meyn LA, Mhlanga F, Chirenje ZM, Hillier SL (2018). Impact of contraceptive initiation on vaginal microbiota. Am J Obstet Gynecol..

[31] Brooks JP, Edwards DJ, Blithe DL, Fettweis JM, Serrano MG, Sheth NU (2017). Effects of combined oral contraceptives, depot medroxyprogesterone acetate and the levonorgestrel-releasing intrauterine system on the vaginal microbiome. Contraception..

[32] Jespers V, Kyongo J, Joseph S, Hardy L, Cools P, Crucitti T (2017). A longitudinal analysis of the vaginal microbiota and vaginal immune mediators in women from sub-Saharan Africa. Sci Rep..

[33] Song S, Acharya K, Zhu J, Deveney C, Walther-Antonio MR, Tetel M, et al. Daily vaginal microbiota fluctuations associated with natural hormonal cycle, contraceptives, diet, and exercise. mSphere [Internet]. 2020;5:e00593-20. Available from: 10.1128/mSphere.00593-20. 10.1128/mSphere.00593-20 PMC7343982 32641429

[34] Mitchell CM, McLemore L, Westerberg K, Astronomo R, Smythe K, Gardella C, et al. Long-term effect of depot medroxyprogesterone acetate on vaginal microbiota, epithelial thickness and HIV target cells. J Infect Dis [Internet]. 2014;210:651–5. Available from: 10.1093/infdis/jiu176. 10.1093/infdis/jiu176 PMC4172039 24652495

[35] Wessels JM, Lajoie J, Cooper MIJH, Omollo K, Felker AM, Vitali D, et al. Medroxyprogesterone acetate alters the vaginal microbiota and microenvironment in women and increases susceptibility to HIV-1 in humanized mice. Dis Model Mech [Internet]. 2019;12:dmm039669. Available from: 10.1242/dmm.039669. 10.1242/dmm.039669 PMC6826019 31537512

[36] Lisa B Haddad, Jennifer H Tang, Nicole L Davis, Athena P Kourtis, Lameck Chinula, Albans Msika, et al. Influence of hormonal contraceptive use and HIV on cervicovaginal cytokines and microbiota in Malawi. mSphere [Internet]. 2023;0:e00585-22. Available from: 10.1128/msphere.00585-22. 10.1128/msphere.00585-22 PMC9942570 36622252

[37] Passmore J-AS, Jaspan HB, Masson L. Genital inflammation, immune activation and risk of sexual HIV acquisition. Curr Opin HIV AIDS [Internet]. 2016;11:156–62. Available from: https://pubmed.ncbi.nlm.nih.gov/26628324. Accessed 18 Dec 2020 10.1097/COH.0000000000000232 PMC6194860 26628324

[38] Liebenberg LJP, Masson L, Arnold KB, Mckinnon LR, Werner L, Proctor E (2017). Genital — systemic chemokine gradients and the risk of HIV acquisition in women. J Acquir Immune Defic Syndr..

[39] Masson L, Passmore J-AS, Liebenberg LJ, Werner L, Baxter C, Arnold KB, Williamson C, Little F, Mansoor LE, Naranbhai V, Lauffenburger DA, Ronacher K, Walzl G, Garrett NJ, Williams BL, Couto-Rodriguez M, Hornig M, Lipkin WI, Grobler A, Abdool Karim SS. Genital inflammation and the risk of HIV acquisition in women. Clinical Infectious Diseases. 2015;61(2):260–9. 10.1093/cid/civ298. 10.1093/cid/civ298 PMC4565995 25900168

[40] McKinnon LR, Liebenberg LJ, Yende-Zuma N, Archary D, Ngcapu S, Sivro A (2018). Genital inflammation undermines the effectiveness of tenofovir gel in preventing HIV acquisition in women. Nat Med..

[41] Mlisana K, Naicker N, Werner L, Roberts L, van Loggerenberg F, Baxter C (2012). Symptomatic vaginal discharge is a poor predictor of sexually transmitted infections and genital tract inflammation in high-risk women in South Africa. J Infect Dis.

[42] Happel A-U, Sivro A, Liebenberg L, Passmore JA, Mitchell CM. Considerations for choosing soluble immune markers to determine safety of novel vaginal products. Front Reprod Health [Internet]. 2022;4. Available from: https://www.frontiersin.org/articles/10.3389/frph.2022.899277. Accessed 18 Nov 2022 10.3389/frph.2022.899277 PMC9580790 36303630

[43] Dabee S, Barnabas SL, Lennard KS, Jaumdally SZ, Gamieldien H, Balle C, et al. Defining characteristics of genital health in South African adolescent girls and young women at high risk for HIV infection. PLoS One [Internet]. 2019;14:e0213975–e0213975. Available from: https://www.ncbi.nlm.nih.gov/pubmed/30947260. Accessed 06 Dec 2019 10.1371/journal.pone.0213975 PMC6448899 30947260

[44] Deese J, Masson L, Miller W, Cohen M, Morrison C, Wang M (2015). Injectable progestin-only contraception is associated with increased levels of pro-inflammatory cytokines in the female genital tract. Am J Reprod Immunol.

[45] Francis SC, Hou Y, Baisley K, van de Wijgert J, Watson-Jones D, Ao TT, Herrera C, Maganja K, Andreasen A, Kapiga S, Coulton GR. Immune activation in the female genital tract: expression profiles of soluble proteins in women at high risk for HIV infection. PloS one. 2016;11(1):e0143109. 10.1371/journal.pone.0143109 PMC4729472 26814891

[46] Morrison CS, Fichorova R, Chen P-L, Kwok C, Deese J, Yamamoto H (2018). A longitudinal assessment of cervical inflammation and immunity associated with HIV-1 infection, hormonal contraception, and pregnancy. AIDS Res Hum Retroviruses.

[47] Huijbregts RPH, Helton ES, Michel KG, Sabbaj S, Richter HE, Goepfert PA (2013). Hormonal contraception and HIV-1 infection: medroxyprogesterone acetate suppresses innate and adaptive immune mechanisms. Endocrinology.

[48] Ngcapu S, Masson L, Sibeko S, Werner L, McKinnon LR, Mlisana K (2015). Lower concentrations of chemotactic cytokines and soluble innate factors in the lower female genital tract associated with the use of injectable hormonal contraceptive. J Reprod Immunol.

[49] Michel KG, Huijbregts RPH, Gleason JL, Richter HE, Hel Z (2015). Effect of hormonal contraception on the function of plasmacytoid dendritic cells and distribution of immune cell populations in the female reproductive tract. J Acquir Immune Defic Syndr.

[50] Tasker C, Pizutelli V, Lo Y, Ramratnam B, Roche NE, Chang TL. Depot medroxyprogesterone acetate administration increases cervical CCR5+CD4+ T cells and induces immunosuppressive milieu at the cervicovaginal mucosa. AIDS [Internet]. 2020;34. Available from: https://journals.lww.com/aidsonline/Fulltext/2020/04010/Depot_medroxyprogesterone_acetate_administration.9.aspx. Accessed 18 Nov 2022 10.1097/QAD.0000000000002475 PMC7337252 31972606

[51] Smith-McCune KK, Hilton JF, Shanmugasundaram U, Critchfield JW, Greenblatt RM, Seidman D, et al. Effects of depot-medroxyprogesterone acetate on the immune microenvironment of the human cervix and endometrium: implications for HIV susceptibility. Mucosal Immunol [Internet]. 2017;10:1270–8. Available from: 10.1038/mi.2016.121. 10.1038/mi.2016.121 PMC5496803 28051087

[52] Achilles SL, Meyn LA, Mhlanga FG, Matubu AT, Stoner KA, Beamer MA, et al. Zim CHIC: a cohort study of immune changes in the female genital tract associated with initiation and use of contraceptives. Am J Reprod Immunol [Internet]. 2020;84:e13287. Available from: 10.1111/aji.13287. 10.1111/aji.13287 PMC7507197 32533883

[53] Omollo K, Lajoie J, Oyugi J, Wessels JM, Mwaengo D, Kimani J, et al. Differential elevation of inflammation and CD4+ T cell activation in Kenyan female sex workers and non-sex workers using depot-medroxyprogesterone acetate. Front Immunol [Internet]. 2021;11. Available from: https://www.frontiersin.org/articles/10.3389/fimmu.2020.598307. Accessed 15 Nov 2022 10.3389/fimmu.2020.598307 PMC7949914 33717049

[54] Molatlhegi RP, Liebenberg LJ, Leslie A, Noel-Romas L, Mabhula A, Mchunu N, et al. Plasma concentration of injectable contraceptive correlates with reduced cervicovaginal growth factor expression in South African women. Mucosal Immunol [Internet]. 2020;13:449–59. Available from: 10.1038/s41385-019-0249-y. 10.1038/s41385-019-0249-y PMC7617771 31896762

[55] Byrne EH, Anahtar MN, Cohen KE, Moodley A, Padavattan N, Ismail N, et al. Association between injectable progestin-only contraceptives and HIV acquisition and HIV target cell frequency in the female genital tract in South African women: a prospective cohort study. Lancet Infect Dis [Internet]. 2016;16:441–8. Available from: 10.1016/S1473-3099(15)00429-6. 10.1016/S1473-3099(15)00429-6 PMC5294917 26723758

[56] •• Radzey N, Harryparsad R, Meyer B, Chen PL, Gao X, Morrison C, et al. Genital inflammatory status and the innate immune response to contraceptive initiation. Am J Reprod Immunol [Internet]. 2022;88:e13542. Available from: 10.1111/aji.13542. **This biomedical ECHO sub-study showed that DMPA-IM use does not increase cervico-vaginal cytokines levels at 1- and 3-month post-contraceptive initiation compared to pre-initiation.** 10.1111/aji.13542 PMC10909525 35394678

[57] Monin L, Whettlock EM, Male V. Immune responses in the human female reproductive tract. Immunology [Internet]. 2020;160:106–15. Available from: 10.1111/imm.13136. 10.1111/imm.13136 PMC7218661 31630394

[58] Alvarez Y, Tuen M, Shen G, Nawaz F, Arthos J, Wolff MJ (2013). Preferential HIV infection of CCR6+ Th17 cells is associated with higher levels of virus receptor expression and lack of CCR5 ligands. J Virol.

[59] Sun H, Kim D, Li X, Kiselinova M, Ouyang Z, Vandekerckhove L (2015). Th1/17 polarization of CD4 T cells supports HIV-1 persistence during antiretroviral therapy. J Virol.

[60] Lajoie J, Tjernlund A, Omollo K, Edfeldt G, Röhl M, Boily-Larouche G, et al. Increased cervical CD4+CCR5+ T cells among Kenyan sex working women using depot medroxyprogesterone acetate. AIDS Res Hum Retroviruses [Internet]. 2018;35:236–46. Available from: 10.1089/aid.2018.0188. Accessed 18 Nov 2022 10.1089/aid.2018.0188 PMC6434599 30585733

[61] Ildgruben AK, Sjöberg IM, Hammarström M-LKC. Influence of hormonal contraceptives on the immune cells and thickness of human vaginal epithelium. Obstet Gynecol [Internet]. 2003;102:571–82. Available from: https://www.sciencedirect.com/science/article/pii/S0029784403006185. Accessed 18 Nov 2022. 10.1016/s0029-7844(03)00618-5 12962945

[62] Edfeldt G, Lajoie J, Röhl M, Oyugi J, Åhlberg A, Khalilzadeh-Binicy B, et al. Regular use of depot medroxyprogesterone acetate causes thinning of the superficial lining and apical distribution of human immunodeficiency virus target cells in the human ectocervix. J Infect Dis [Internet]. 2022;225:1151–61. Available from: 10.1093/infdis/jiaa514. 10.1093/infdis/jiaa514 PMC8974825 32780807

[63] Chandra N, Thurman AR, Anderson S, Cunningham TD, Yousefieh N, Mauck C, et al. Depot medroxyprogesterone acetate increases immune cell numbers and activation markers in human vaginal mucosal tissues. AIDS Res Hum Retroviruses [Internet]. 2012;29:592–601. Available from: 10.1089/aid.2012.0271. 10.1089/aid.2012.0271 PMC3581024 23189932

[64] Li L, Zhou J, Wang W, Huang L, Tu J, Baiamonte L, et al. Effects of three long-acting reversible contraceptive methods on HIV target cells in the human uterine cervix and peripheral blood. Reprod Biol Endocrinol [Internet]. 2019;17:26. Available from: 10.1186/s12958-019-0469-8. 10.1186/s12958-019-0469-8 PMC6387540 30795774

[65] Carias A, Hope T (2019). Barriers of mucosal entry of HIV/SIV. Curr Immunol Rev..

[66] Molatlhegi RP, Liebenberg LJ, Leslie A, Noel-Romas L, Mabhula A, Mchunu N (2020). Plasma concentration of injectable contraceptive correlates with reduced cervicovaginal growth factor expression in South African women. Mucosal Immunol..

[67] Keller MJ, Guzman E, Hazrati E, Kasowitz A, Cheshenko N, Wallenstein S (2007). PRO 2000 elicits a decline in genital tract immune mediators without compromising intrinsic antimicrobial activity. AIDS.

[68] Haddad LB, Swaims-Kohlmeier A, Mehta CC, Haaland RE, Brown NL, Sheth AN (2020). Impact of etonogestrel implant use on T-cell and cytokine profiles in the female genital tract and blood. PLoS One..

[69] Birse KD, Romas LM, Guthrie BL, Nilsson P, Bosire R, Kiarie J (2017). Genital injury signatures and microbiome alterations associated with depot medroxyprogesterone acetate usage and intravaginal drying practices. J Infect Dis.

[70] Vincent AJ, Zhang J, Ostör A, Rogers PAW, Affandi B, Kovacs G (2002). Decreased tissue inhibitor of metalloproteinase in the endometrium of women using depot medroxyprogesterone acetate: a role for altered endometrial matrix metalloproteinase/tissue inhibitor of metalloproteinase balance in the pathogenesis of abnormal ute. Hum Reprod.

[71] Zalenskaya IA, Chandra N, Yousefieh N, Fang X, Adedipe OE, Jackson SS (2018). Use of contraceptive depot medroxyprogesterone acetate is associated with impaired cervicovaginal mucosal integrity. J Clin Invest..

[72] Calla NEQ, Miguel RDV, Boyaka PN, Hall-stoodley L, Kaur B, Trout W (2016). Medroxyprogesterone acetate and levonorgestrel increase genital mucosal permeability and enhance susceptibility to genital herpes simplex virus type 2 infection. Mucosal Immunol..

[73] Goldfien GA, Barragan F, Chen J, Takeda M, Irwin JC, Perry J, et al. Progestin-containing contraceptives alter expression of host defense-related genes of the endometrium and cervix. Reprod Sci [Internet]. 2015;22:814–28. Available from: 10.1177/1933719114565035. 10.1177/1933719114565035 PMC4565478 25634912

[74] Edfeldt G, Lajoie J, Röhl M, Oyugi J, Åhlberg A, Khalilzadeh-Binicy B, Bradley F, Mack M, Kimani J, Omollo K, Wählby C. Regular use of depot medroxyprogesterone acetate causes thinning of the superficial lining and apical distribution of human immunodeficiency virus target cells in the human ectocervix. The Journal of Infectious Diseases. 2022;225(7):1151–61. 10.1093/infdis/jiaa514 PMC8974825 32780807

[75] Bahamondes L, Trevisan M, Andrade L, Marchi NM, Castro S, Díaz J, et al. The effect upon the human vaginal histology of the long-term use of the injectable contraceptive Depo-Provera. Contraception [Internet]. 2000;62:23–7. Available from: 10.1016/S0010-7824(00)00132-3. 10.1016/s0010-7824(00)00132-3 11024225

[76] Slifer ZM, Blikslager AT. The integral role of tight junction proteins in the repair of injured intestinal epithelium. Int J Mol Sci [Internet]. 2020;21. Available from: https://www.mdpi.com/1422-0067/21/3/972. Accessed 16 Feb 2023 10.3390/ijms21030972 PMC7036844 32024112

[77] Wu G, Grassi P, MacIntyre DA, Molina BG, Sykes L, Kundu S, et al. N-glycosylation of cervicovaginal fluid reflects microbial community, immune activity, and pregnancy status. Sci Rep [Internet]. 2022;12:16948. Available from: 10.1038/s41598-022-20608-7. 10.1038/s41598-022-20608-7 PMC9551102 36216861

[78] Agarwal K, Choudhury B, Robinson LS, Allsworth JE, Lewis AL, Lewis WG. Resident microbes shape the vaginal epithelial glycan landscape. medRxiv [Internet]. 2022;2022.02.23.22271417. Available from: http://medrxiv.org/content/early/2022/02/24/2022.02.23.22271417.abstract. Accessed 16 Feb 2023 10.1126/scitranslmed.abp9599 PMC11419735 38019934

[79] Barker EK, Malekinejad M, Merai R, Lyles CM, Sipe TA, DeLuca JB, et al. Risk of human immunodeficiency virus acquisition among high-risk heterosexuals with nonviral sexually transmitted infections: a systematic review and meta-analysis. Sex Transm Dis [Internet]. 2022;49. Available from: https://journals.lww.com/stdjournal/Fulltext/2022/06000/Risk_of_Human_Immunodeficiency_Virus_Acquisition.1.aspx. Accessed 18 Nov 2022 10.1097/OLQ.0000000000001601 PMC9133024 35034049

[80] Looker KJ, Rönn MM, Brock PM, Brisson M, Drolet M, Mayaud P, et al. Evidence of synergistic relationships between HIV and human papillomavirus (HPV): systematic reviews and meta-analyses of longitudinal studies of HPV acquisition and clearance by HIV status, and of HIV acquisition by HPV status. J Int AIDS Soc [Internet]. 2018;21:e25110. Available from: 10.1002/jia2.25110. 10.1002/jia2.25110 PMC5989783 29873885

[81] Looker KJ, Welton NJ, Sabin KM, Dalal S, Vickerman P, Turner KME, et al. Global and regional estimates of the contribution of herpes simplex virus type 2 infection to HIV incidence: a population attributable fraction analysis using published epidemiological data. Lancet Infect Dis [Internet]. 2020;20:240–9. Available from: 10.1016/S1473-3099(19)30470-0. 10.1016/S1473-3099(19)30470-0 PMC6990396 31753763

[82] McCarthy KJ, Gollub EL, Ralph L, van de Wijgert J, Jones HE. Hormonal contraceptives and the acquisition of sexually transmitted infections: an updated systematic review. Sex Transm Dis [Internet]. 2019;46. Available from: https://journals.lww.com/stdjournal/Fulltext/2019/05000/Hormonal_Contraceptives_and_the_Acquisition_of.3.aspx. Accessed 15 Nov 2022 10.1097/OLQ.0000000000000975 30628946

[83] Deese J, Pradhan S, Goetz H, Morrison CS (2018). Contraceptive use and the risk of sexually transmitted infection: systematic review and current perspectives. Open Access J Contracept..

